# *BRAF* mutation correlates with worse local–regional control following radiation therapy in patients with stage III melanoma

**DOI:** 10.1186/s13014-021-01903-5

**Published:** 2021-09-18

**Authors:** Adam R. Wolfe, Priyanka Chablani, Michael R. Siedow, Eric D. Miller, Steve Walston, Kari L. Kendra, Evan Wuthrick, Terence M. Williams

**Affiliations:** 1 Department of Radiation Oncology, The University of Arkansas for Medical Sciences, The Winthrop P. Rockefeller Cancer Institute, Little Rock, AR USA; 2 Division of Hematology-Oncology, Department of Internal Medicine, University of Chicago, Chicago, IL USA; 3 Department of Radiation Oncology, The Ohio State University Wexner Medical Center, Columbus, OH USA; 4 Division of Hematology-Oncology, Department of Internal Medicine, The Ohio State University Wexner Medical Center, Columbus, OH USA; 5 Department of Radiation Oncology, Moffitt Cancer Center, Tampa, FL USA; 6 Department of Radiation Oncology, City of Hope National Medical Center, 1500 E. Duarte Road, Duarte, CA 91010 USA

## Abstract

**Background:**

In patients with stage III melanoma, the use of adjuvant radiation therapy (RT) after lymph node dissection (LND) may be currently considered in selected high-risk patients to improve tumor control. Melanomas harbor *BRAF* mutations *(BRAF*+) in 40–50% of cases, the majority of which are on the V600E residue. This study sought to compare the clinical outcomes after RT between patients with *BRAF*+ and *BRAF*− melanoma.

**Methods:**

This was a retrospective review of 105 Stage III melanoma patients treated at our institution with LND followed by adjuvant RT from 2006 to 2019. *BRAF* mutational status was determined on the primary skin or nodal tissue samples from all patients. We compared characteristics of the *BRAF*+ and *BRAF*− groups using Fisher’s exact test and Wilcoxon rank sum test and performed univariate and multivariate analysis using Kaplan–Meier estimates, log-rank tests, and Cox proportional hazards modeling with the clinical outcomes of local–regional lymph node control, distant metastasis-free survival (DMFS), recurrence-free survival (RFS), and overall survival (OS).

**Results:**

Fifty-three (50%) patients harbored a *BRAF* mutation (92%, pV600E). *BRAF*+ patients were younger and had primary tumors more commonly found in the trunk vs head and neck compared to *BRAF*- patients (*p* < 0.05). The 5 year local–regional control in the *BRAF* + patients was 60% compared to 81% in the *BRAF*- patients (HR 4.5, 95% CI 1.3–15.5, *p* = 0.02). There were no significant differences in 5-year DMFS, RFS, and OS rates between the two *BRAF* patient groups. The presence of 4 or more positive LNs remained a significant prognostic factor for local–regional lymph node control, RFS, and OS in multivariate analysis.

**Conclusions:**

Stage III melanoma patients with *BRAF* mutation treated with adjuvant RT had > 4 times increased risk of local recurrence or regional lymph node recurrence. These results could be useful for adjuvant RT consideration in lymph node positive melanoma patients and supports other data that BRAF mutation confers radiation resistance.

## Introduction

In patients with melanoma, a common site of metastasis is the regional lymph node (LN) basin, and patients with clinically positive regional LNs often undergo therapeutic lymph node dissection (LND) to reduce the risk of nodal recurrence. There is a significant risk of recurrence in stage III (node-positive) melanoma patients even after LND, and 5-year survival rates range from 30 to 90% depending on features such as number of LNs involved, extracapsular extension, Breslow thickness, ulceration, etc. [1, 2]. One treatment option to improve both local and regional lymph-node control following LND is adjuvant radiation therapy (RT). Adjuvant (post-operative) RT is considered for patients who are at high-risk for local and regional LN recurrence [2]. These high-risk criteria are based on the phase III ANZMTG 01.02/TROG 02.01 trial which randomized patients with clinically or pathologically positive LNs who had undergone lymphadenectomy to adjuvant radiation or observation and the trial results showed improved nodal relapse-free survival in the adjuvant RT arm compared to the observation group [3].

The *BRAF* gene encodes a protein kinase (MAPK) that regulates cellular growth and proliferation in tumor cells [4]. Mutations in *BRAF* can cause constitutive activation of the MAPK pathway. The most common mutation in the *BRAF* gene found in melanoma patients is the substitution of a glutamic acid for a valine at the amino acid 600 position (V600E). Approximately 50% of melanomas harbor activating *BRAF* mutations, with 70–90% of these mutations being V600E [5]. More recently, stage III melanoma patients with *BRAF* mutations may receive adjuvant treatment with either the combination *BRAF* and MEK inhibitors, dabrafenib and trametinib or immunotherapy [6, 7]. *BRAF* wild-type patients may receive adjuvant immunotherapy with one of the programmed cell death protein 1 (PD-1) inhibitors, nivolumab or pembrolizumab, or the cytotoxic T-lymphocyte-associated protein 4 (CTLA-4) inhibitor, ipilimumab [8–10]. The benefit of adjuvant RT based on *BRAF* mutational status is unclear. In addition, preclinical data suggest that *BRAF* mutation increases radiation resistance in both anaplastic thyroid cancer [11] and melanoma [12]. In this study, we aimed to investigate the outcomes of adjuvant RT on local and regional LN control along with other clinical outcomes in patients with *BRAF*+ and *BRAF*− melanoma. Given the controversial nature of adjuvant RT, we sought to determine whether either of these subpopulations benefits more or less from adjuvant RT, and thereby would be more or less suitable for this therapy.

## Methods

### Patient Selection

This was an institutional review board (IRB) approved retrospective chart review. We reviewed the electronic medical records of patients with clinical or radiographic evidence of LN basin metastasis stage III melanoma who went on to receive a therapeutic LND and adjuvant RT at our institution from 2006 to 2018. Patients were excluded if they received simultaneous systemic chemotherapy at the time of adjuvant RT or if they had received previous RT in the nodal field. Ultimately, 105 patients were included in the final analysis who met the above criteria and had BRAF mutational status performed. For each patient, we collected data related to demographics, staging, pathology, *BRAF* mutational status, treatment, and outcomes.

### Staging and treatment

Clinical staging was done with a physical exam and CT and/or MRI imaging. Pathologic staging was done after LND according to the American Joint Committee on Cancer (AJCC 7^th^ ED) guidelines based on tumor invasion, LN involvement, and metastasis. All patients underwent LND prior to radiation therapy. The majority of patients received external beam radiation therapy based on two fractionation schemes: 30 Gy over 5 fractions (6 Gy/fraction) or 48 Gy over 20 fractions (2.4 Gy/fraction). For patients with the primary site of disease in the head and neck, standard fractionation schemes of 1.8–2 Gy/fraction, generally to 50–66 Gy were utilized.

### Pathologic analysis

Pathologists specializing in melanoma at our institution examined the LND specimens using AJCC criteria. The features they assessed included tumor depth of invasion, ulceration in primary, site of LNs, number of LNs positive, number of LNs dissected, size of largest melanoma deposit, and extracapsular or extranodal invasion.

*BRAF* mutational status was determined from chart review and was obtained through either a PCR-based sequencing assay or FoundationOne® genomic analysis that was requested by the provider team. Sequencing was performed on either the initial biopsy, re-excision specimen, or LND specimen. In the majority of cases, this PCR-based *BRAF* assay was ordered by the treating oncologist for clinical purposes. For PCR testing, genomic DNA was extracted from tumor tissue (fresh frozen, or formalin fixed paraffin embedded tissue) and Exon 15 of the BRAF gene was amplified by polymerase chain reaction (PCR), and subsequently a SNPlex and/or direct nucleotide sequencing by PCR-based cycle sequencing method was used to evaluate for point mutation in BRAF codon 600. Four of the 53 *BRAF*+ patients were found on FoundationOne® testing to have a non-V600E mutation: V600K (2), (1) G466R (1), and K601N (1).

### Statistical analysis

We compared characteristics between patients who were *BRAF*+ and *BRAF*− using the Fisher’s exact test for categorical variables and Wilcoxon rank sum test for continuous variables. We evaluated clinical outcomes including cumulative incidence of combined local failure and regional LN failure distant metastasis-free survival (DMFS), recurrence-free survival (RFS), and overall survival (OS). Local and/or regional failures were defined as the interval from the date of LND to the date of recurrence found on clinical exam or CT imaging. Local relapse or regional lymph-node field relapse was defined as a first relapse either isolated or concurrent with relapse at any other site. Time to lymph-node field relapse was censored by relapse in other sites, death, the cutoff date, and loss to follow-up. DMFS was defined as the interval from the date of LND to the date of distant failure reported on clinical imaging or death from any cause. RFS was the interval from LND to local–regional recurrence or distant failure, whichever occurred first or death from any cause. OS was the time from LND to death from any cause. Patients who were alive at the last clinical follow-up visit were censored from the OS analysis at that time.

We performed a univariate analysis of variables associated with local and LN recurrence, distant failures, RFS, and OS using Kaplan–Meier estimates and log-rank tests. We then performed a multivariate analysis of variables associated with local and regional LN control, DMFS, RFS, and OS using Cox proportional hazards modeling. Statistical analysis was done using SPSS Statistics 27. (IBM SPSS, Armonk, New York) and MedCalc for Windows, version 19.4 (MedCalc Software, Ostend, Belgium).

## Results

### Patient and clinical characteristics

One hundred and five patients with Stage III melanoma and available *BRAF* mutational status met the inclusion criteria for our study. The demographic, clinical, pathological, and treatment characteristics are displayed in Table 1. In this cohort, 53 (50%) patients were found to be positive for a *BRAF* mutation (*BRAF*+) and 52 (50%) were negative for a *BRAF* mutation (*BRAF*-). The *BRAF*+ patients were younger compared to *BRAF*- patients (median age 51.9 vs 61.7, *p* = 0.08). There was no difference in the genders between the two groups (68% and 65% male). We found a statistically significant difference between the primary tumor sites between the two groups. *BRAF*+ patients were more likely to have tumors located in the trunk compared to the *BRAF*- patients (34% vs 11%) while the *BRAF*- patients more commonly had primary tumors located in the head and neck regions (43% vs 19%, *p* = 0.02 between all groups).

**Table 1:** Demographic, clinical, pathological, and treatment characteristics of the *BRAF*+ and *BRAF*− melanoma groups

Variable	*BRAF*+ (n = 53)	*BRAF*− (n = 52)	*P*
**Age (years)**			
≤ 50	23 (43%)	10 (19%)	**0.01**
> 50	30 (57%)	42 (81%)	
**Sex**			
Male	36 (68%)	34 (65%)	0.89
Female	17 (32%)	18 (35%)	
**Site of primary**			
Head and Neck	10 (19%)	23 (43%)	
Trunk	18 (34%)	6 (11%)	**0.02**
Arm	10 (19%)	9 (17%)	
Leg	11 (21%)	9 (17%)	
Unknown (found only in LNs)	4 (8%)	5 (9%)	
**Site of LND**			
Cervical	7 (19%)	14 (44%)	0.09
Axillary	18 (50%)	11 (34%)	
Inguinal	11 (31%)	7 (22%)	
**Depth of invasion at biopsy**			
T1 (≤ 1 mm)	3 (6%)	1 (2%)	
T2 (> 1–2 mm)	4 (8%)	2 (4%)	0.58
T3 (> 2–4 mm)	5 (10%)	8 (16%)	
T4 (> 4 mm)	16 (32%)	13 (27%)	
Unknown	22 (44%)	25 (51%)	
**Ulceration in biopsy**			
No	17 (32%)	11 (21%)	0.38
Yes	19 (36%)	19 (37%)	
Unknown	17 (32%)	22 (42%)	
**# of Mitosis in Biopsy (mm**^**2**^**)**			
0–2	10 (19%)	4 (8%)	**0.04**
3–5	6 (11%)	11 (21%)	
> 5	20 (38%)	11 (21%)	
Unknown	17 (32%)	26 (50%)	
**# of LNs positive in LND**			
1 (N1)	8 (35%)	8 (42%)	0.86
2 or 3 (N2)	14 (61%)	10 (53%)	
4 + (N3)	1 (4%)	1 (5%)	
**ECE found in LND**			
Yes	20 (56%)	14 (44%)	0.47
No	16 (44%)	18 (56%)	

### Pathological characteristics

We found that there were no significant differences between the two groups in regard to depth of invasion of the tumor on biopsy or presence of ulceration on the biopsy pathology reports. The percentage of patients with the number of tumor mitoses per mm^2^ greater than 5 was higher in the *BRAF*+ group (38% vs 21%, *p* = 0.036). All patients underwent a LND, and the number of positive LNs was not found to be different between the two groups (median 2.0 for both groups). In addition, there were no significant differences in presence of ECE (*p* = 0.47) or size of the largest tumor deposit within the LND specimens (*p* = 0.12).

### Treatment characteristics

The median time between the LND and the start of RT was not significantly different between the two groups (median 4.27 for *BRAF*+ vs 4.81 months for *BRAF*−, *p* = 0.1). The total dose of RT delivered was also not significantly different between the groups (median 30 Gy vs 36 Gy, p = 0.55). The use of adjuvant systemic therapy was also non-significant (60% vs 50%, *p* = 0.33). With regard to systemic therapy, 58 (55%) patients received adjuvant systemic therapy, with therapies including interferon (21), dendritic vaccine (4), ipilimumab (12), pembrolizumab (2), nivolumab (5), dabrafenib plus trametinib (2), and temozolomide (2) delivered after the course adjuvant RT. Patients then underwent follow-up exams and re-staging scans at intervals determined by the treatment team.

### Survival outcomes

**Fig. 1:**
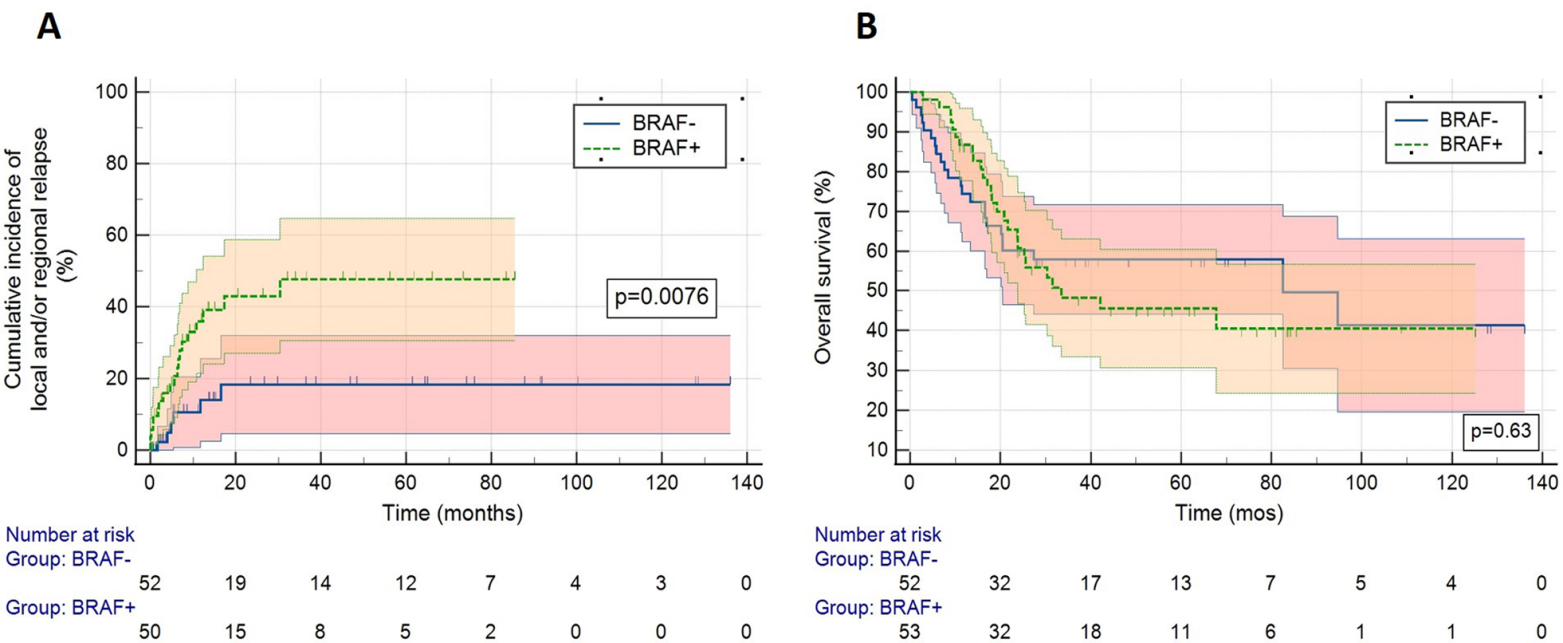
**a** Cumulative local and/or regional (LN) relapse. **b** Overall survival stratified based on patient *BRAF* mutational status

The median follow-up was 23.8 and 27.6 months in the *BRAF*+ and *BRAF*− groups, respectively. The 5-year local and regional LN control rate was improved in the *BRAF*− patients compared to the *BRAF*+ patients. The total number of local or regional (LN) failures was 18 and 7 in the *BRAF* + and *BRAF*- groups, respectively. The 5-year local–regional LN failure rate in the *BRAF* + was 48% (SD 7.9%) vs 19% (SD 6.6%) in the *BRAF*- patients (*p* = 0.0076) (Fig. 1a). All of the local–regional LN failures occurred within the first three years after RT. There was no significant difference in DMFS between the two groups, with the 5-year DMFS of 38% (SD 7%) in the *BRAF*+ group vs 34% (SD 8%) in the *BRAF*- group, *p* = 0.79. RFS was also not significantly different between the two groups. The 5-year RFS was 23% vs 36% in the *BRAF*+ and *BRAF*− patients respectively (*p* = 0.38). Finally, the 5-year OS in the *BRAF*+ patients was 46% (SD 8%) compared to 59% (SD 7%) in the *BRAF*- patients (*p* = 0.63) **(**Fig. 1b).

### Univariate and multivariate analysis

We performed univariate analysis of variables associated with local–regional LN control, DMFS, RFS, and OS (Table 2). The only two variables found to significantly correlate with worse local–regional control were *BRAF*+ status (HR 2.7 95% CI 1.1.6.5 *p* = 0.03) and ≥ 4 LNs positive (HR 3.3, 95% CI 1.4–8.2 *p* = 0.01). There were no variables significantly correlated with DMFS, although the number of positive LNs and use of adjuvant systemic therapy trended toward significance (*p* < 0.1). For RFS analysis, the two variables that were found to be statistically significant were ≥ 4 LNs (HR 1.9, *p* = 0.03) and the use of adjuvant systemic therapy (HR 0.61, *p* = 0.04). Lastly, only ≥ 4 LNs predicted for worse OS (HR 2.3, *p* = 0.02).

**Table 2:** Univariate analysis of clinical outcomes

Variable	Local–Regional (LN) Control	*P*	DMFS	*P*	RFS	*P*	OS	*P*
***BRAF* status**								
Negative	–	**0.03**	–	0.89	–	0.45	–	0.72
Positive	2.7 (1.1–6.5)		0.96 (0.58–1.6)		1.20 (0.75–1.9)		1.11 (0.63–2.0)	
**Gender**								
Male	–	0.12	–	0.89	–	0.87	–	0.87
Female	0.46 (0.17–1.2)		0.93 (0.55–1.6)		1.04 (0.63–1.7)		0.95 (0.52–1.7)	
**Age**								
≤ 50 yrs	–	0.54	–	0.55	–	0.60	–	0.22
< 50 yrs	0.77 (0.33–1.8)		1.12 (0.69–2.0)		1.14 (0.69–1.9)		1.46 (0.79–2.7)	
**Location of primary**								
Other	–	0.99	–	0.57	–	0.71	–	0.23
Trunk	1.01 (0.35–2.9)		0.80 (0.38–1.7)		0.86 (0.43–1.8)		0.57 (0.23–1.4)	
**Depth of invasion**								
≤ 2 mm	–	0.94	–	0.70	–	0.91	–	0.51
< 2 mm	1.04 (0.43–2.5)		1.11 (0.64–1.9)		0.97 (0.58–1.6)		0.82 (0.45–1.5)	
**Ulceration of primary**								
No	–	0.95	–	0.67	–	0.89	–	0.61
Yes	1.04 (0.36–2.9)		1.15 (0.61–2.1)		0.96 (0.53–1.7)		1.20 (0.60–2.4)	
**# of Mitosis in primary**								
≤ 5	–	0.74	–	0.83	–	0.86	–	0.72
> 5	1.20 (0.41–3.4)		1.1 (0.56–2.0)		1.01 (0.58–1.9)		1.13 (0.57–2.3)	
**# of LNs positive**								
< 4	–	**0.01**	–	0.09	–	**0.03**	–	**0.02**
≥ 4	3.34 (1.4–8.2)		1.72 (0.92–3.2)		1.88 (1.1–3.4)		2.26 (1.19–4.3)	
**ECE**								
No	–	0.85	–	0.60	–	0.87	–	0.79
Yes	0.91 (0.35–2.4)		1.18 (0.62–2.2)		0.95 (0.52–1.7)		1.09 (0.56–2.1)	
**Size of LN deposit**								
≤ 30 mm	–	0.11	–	0.32	–	0.47	–	0.93
> 30 mm	0.41 (0.14–1.2)		1.35 (0.75–2.4)		1.23 (0.70–2.1)		0.97 (0.51–1.9)	
**RT dose**								
≤ 30 Gy	–	0.99	–	0.98	–	0.72	–	0.57
> 30 Gy	0.99 (0.46–2.2)		0.99 (0.60–1.6)		1.1 (0.68–1.8)		0.85 (0.48–1.5)	
**Time from LND to RT**								
≤ 3 mos	–	0.55	–	0.88	–	0.76	–	0.81
> 3 mos	0.54 (0.7–4.0)		1.08 (0.39–3.0)		0.85 (0.30–2.4)		1.14 (0.41–3.2)	
**Adjuvant systemic Tx**								
No	–	0.46	–	0.07	–	**0.04**	–	0.25
Yes	0.74 (0.34–1.6)		0.63 (0.38–1.0)		0.61 (0.4–0.99)		0.72 (0.41–1.3)	

We next included *BRAF* status and the variables from the univariate analysis with *p* values < 0.1 into the multivariate analysis (Table 3). After including both *BRAF* status and number of LNs positive in the local–regional LN control multivariate analysis, both of these variables remained independently significant for worse local–regional LN control (*BRAF* + HR 4.5, *p* = 0.02, ≥ 4 LNs HR 2.8, *p* = 0.03). *BRAF* status, number of LNs positive, and use of adjuvant systemic therapy were included in the multivariate analysis for DMFS and RFS. None of these variables crossed the significance threshold for DMFS. However, both ≥ 4 positive LNs and use of adjuvant systemic therapy were significantly correlated with RFS (≥ 4 LNs HR, 1.87 *p* = 0.04, adjuvant systemic therapy HR 0.57, *p* = 0.04). Lastly, ≥ 4 positive LNs predicted for worse OS on the multivariate analysis after inclusion of *BRAF* status (HR 2.3, *p* = 0.01).

**Table 3:** Multivariate analysis of clinical outcomes

Variable	Local–Regional (LN) Control	*P*	DMFS	*P*	RFS	*P*	OS	*P*
***BRAF* status**								
Negative	–	**0.02**	–	0.94	–	0.46	–	0.94
Positive	4.49 (1.3–15.5)		0.98 (0.55–1.7)		1.23 (0.71–2.1)		1.02 (0.55–1.9)	
**# of LNs positive**								
< 4	–	**0.03**	–	0.07	–	**0.04**	–	**0.01**
≥ 4	2.79 (1.1–6.9)		1.80 (0.95–3.4)		1.87 (1.0–3.4)		2.26 (1.18–4.3)	
**Adjuvant systemic Tx**								
No			–	0.09	–	**0.04**		
Yes			0.62 (0.35–1.1)		0.57 (0.3–0.96)			

## Discussion

In this retrospective single institutional study, we found stage III melanoma patients treated with LND and adjuvant RT whose tumor exhibited a *BRAF* mutation had significantly worse local–regional control compared to patients with *BRAF* negative (wild-type) tumors. We found no differences in the other clinical outcomes evaluated, including DMFS, RFS, and OS.

Adjuvant RT may be considered in patients who have radiographically, clinically or pathologically positive nodes and meet certain criteria for high-risk for local recurrence. The benefit of RT must be weighed against potential toxicities. These high-risk criteria are based on the phase III ANZMTG 01.02/TROG 02.01 trial that randomized patients to adjuvant RT or observation after LND with the following criteria: LDH < 1.5 times the upper limit of normal, as well as ≥ 1 parotid, ≥ 2 cervical or axillary, or ≥ 3 groin positive nodes, a maximum nodal diameter of ≥ 3 cm in the neck, ≥ 4 cm in the axilla or groin, or nodal extracapsular extension [13]. After a median follow-up of 73 months, this phase III trial showed improved regional LN control in the adjuvant RT arm compared to the observation group [3]. Twenty one percent of patients had nodal relapses in the adjuvant RT group compared to 36% in the observation group (HR 0.52 [95% CI 0.31–0.88]; *p* = 0.023). However, there were no significant differences in relapse-free survival or OS between the two groups. Additionally, adjuvant RT was associated with long-term toxic effects such as pain and fibrosis of the skin and subcutaneous tissues. Further, there was a significant increase in lower limb volumes after adjuvant radiotherapy (difference of 7.3% [95% CI 1.5–13.1]; *p* = 0.014) [13].

Similar results to this phase III trial have also been found in multiple retrospective studies, demonstrating an improvement in local control with adjuvant RT, but not long-term survival [14–17]. One retrospective study that looked at 615 patients (509 who received adjuvant RT and 106 who did not), did show that receiving adjuvant RT was significantly associated with improved RFS on multivariate analysis [18]. However, in a study comparing patients from the National Cancer Database (NCDB) who received adjuvant RT after LND versus those who did not, the authors did not find adjuvant RT to be significantly associated with OS on multivariate analysis [17]. The lack of a clear survival benefit from adjuvant RT, compounded with its additional toxicities and costs, has limited its use in many centers. However, adjuvant RT is still considered in selected high-risk patients in the latest NCCN guidelines [19]. The results of this study potentially identify a subgroup of high-risk patients where perhaps adjuvant RT should be further evaluated (e.g. omission versus local/regional treatment intensification).

Over the past couple of decades, there has been a dramatic change in systemic therapies for melanoma. This change has been underscored by a better understanding of the disease’s genetic and immunologic underpinnings. One of the major advancements in our understanding is the role that the mitogen-activated protein kinase (MAPK) signal transduction pathway plays in the pathogenesis of melanoma. The primary activating mutation implicated in this pathway is in the *BRAF* gene [4, 20]. *BRAF* mutations are found in about 40–60% of melanomas, with about 80% occurring as a V600E mutation, 5–30% as V600K mutations, and the rest other rare mutations [21–23]. *BRAF* mutation has been associated as a poor prognostic factor in other cancer types, for example in papillary thyroid cancer [24]. *BRAF* mutation was associated with worse OS in the Medical Research Council Fluorouracil, Oxaliplatin and Irinotecan: Use and Sequencing (MRC FOCUS) rectal trial, with a hazard ratio [HR] of 1.40 (95% CI, 1.20 to 1.65; *p* < 0.0001) [25]. In melanoma, a French institutional cohort of Stage III melanoma patients of which 40% had confirmed *BRAF*+ mutational status showed *BRAF* mutant patients had significantly worse overall survival and distant metastasis-free survival. However, there was no data on the use of adjuvant RT available in this report [26]. An unanswered question is the potential benefit of adjuvant radiation in combination with BRAF and MEK inhibitors in resected BRAF mutated patients. In the COMBI-AD trial, patients with stage III melanoma and BRAF V600E or V600K mutations were randomized to receive 12 months oral dabrafenib plus trametinib or two matched placebos without radiation. ^7^ At the five-year analysis, patients in the treatment arm had significantly improved RFS (hazard ratio for relapse or death, 0.51; 95% CI, 0.42 to 0.61). Importantly, patients who received the combination treatment had roughly half the local/regional relapse events as the first site of relapse compared to the placebo arm (14% vs 26%).^7^ Whether adding radiation could further improve local and regional (LN) control rates in these patients has not been studied in clinical trials.

There is emerging pre-clinical and clinical evidence that *BRAF* or upstream *KRAS* mutations in cancer cells results in heightened radiation resistance. Pre-clinical studies revealed that KRAS inhibition through silencing with siRNAs or pharmaceutical inhibition of farnesylation of KRAS led to radiosensitization of tumor cells [27, 28]. In clinical studies, patients with Stage I non-small cell lung cancer (NSCLC) or liver metastasis treated with ablative radiation (i.e. stereotactic body radiation therapy, SBRT) whose tumors were *KRAS* mutant had worse local control compared to patients with *KRAS* wild-type tumors [29, 30]. Furthermore, a pre-clinical study recently showed that *BRAF* V600E + thyroid cancer cell lines displayed higher resistance to radiation, and forced expression of a *BRAF* V600E mutation into wild-type *BRAF* thyroid cancer cells resulted in increased radiation resistance in vitro [11]. In this study, targeted inhibition of oncogenic *BRAF* with vemurafenib potently radiosensitized *BRAF* mutant thyroid cancer cells in vitro and in vivo. In melanoma cells specifically, Sambade et al. showed treatment of *BRAF* mutated cell lines with the BRAF inhibitor, vemurafenib, in combination with radiation resulted in radiosensitization through an increase in G1 arrest [12].

Our study has some limitations such as the inherent biases in all retrospective studies. Although we found a difference in local and regional recurrences in the *BRAF* mutated patients, this did not impact other clinical outcomes such as RFS or OS. Another limitation was related to the treatment era of most of the study patients, when immunotherapy and molecularly-targeted agents were not standardly like the current era. Additionally, there was no standardization of adjuvant treatment decisions including radiation dose and fractionation, or systemic agent usage at our institution. Finally, changing standards of care with regard to completion lymph node dissection (e.g. related to the results of the MSLT2 trial) make it more challenging to interpret our results in the context of current clinical care.

The use of adjuvant RT in stage III melanoma is somewhat controversial in the immunotherapy era. The RFS in the EORTC 1325 and EORTC 18071 trials were significantly improved, but local–regional recurrence was still 12–15% [10, 31]. We argue that a more selective delivery of adjuvant RT should be considered in patients based on the results of this study. Without strong randomized evidence, the future analysis of prospectively collected multi-institutional and multi-national data could result in high quality evidence as a surrogate for performing a phase III trial, as is being studied in non-melanoma skin cancers through the SKIN-COBRA large database project [32].

## Conclusions

In this study, we found an increased rate of local–regional recurrence after adjuvant RT in patients whose tumors are *BRAF*+ compared to *BRAF-*. We speculate that this might relate to increased radiation resistance in tumors harboring a *BRAF* mutation, or perhaps a more aggressive biology of stage III disease driven by mutant *BRAF*. We also found the number of LNs positive was a factor significantly associated with worse local–regional control and OS on multivariate analysis, which is consistent with results of previous studies [33–35]. Given the small sample size of this study and other limitations, a larger study is needed to confirm the association of *BRAF* mutation with decreased local–regional control after RT. However, our findings suggest that caution should be exercised before recommending adjuvant RT to the nodal basin for patients at high risk of recurrence whose tumors are *BRAF*+, and that novel therapeutic strategies to reverse potential radiation resistance associated with BRAF mutation should be explored.

## Data Availability

All data generated or analyzed during this study are included in the published article.
